# The impact of caesarean section on breastfeeding initiation, duration and difficulties in the first four months postpartum

**DOI:** 10.1186/s12884-016-0876-1

**Published:** 2016-04-26

**Authors:** Amy J. Hobbs, Cynthia A. Mannion, Sheila W. McDonald, Meredith Brockway, Suzanne C. Tough

**Affiliations:** Cumming School of Medicine, Department of Community Health Sciences, University of Calgary, TRW Building 3rd Floor, 3280 Hospital Drive NW, Calgary, Alberta T2N 4Z6 Canada; Faculty of Nursing, University of Calgary, 2800 University Way NW, Calgary, AB T2N1N4 Canada; Cumming School of Medicine, Department of Paediatrics, University of Calgary, 2888 Shaganappi Trail NW, Calgary, Alberta T3B 6A8 Canada

**Keywords:** Caesarean section, Mode of birth, Vaginal delivery, Breastfeeding, Postpartum

## Abstract

**Background:**

The caesarean section (c-section) rate in Canada is 27.1 %, well above the 5–15 % of deliveries suggested by the World Health Organization in 2009. Emergency and planned c-sections may adversely affect breastfeeding initiation, milk supply and infant breastfeeding receptivity compared to vaginal deliveries. Our study examined mode of delivery and breastfeeding initiation, duration, and difficulties reported by mothers at 4 months postpartum.

**Methods:**

The All Our Babies study is a prospective pregnancy cohort in Calgary, Alberta, that began in 2008. Participants completed questionnaires at <25 and 34–36 weeks gestation and approximately 4 months postpartum. Demographic, mental health, lifestyle, and health services data were obtained. Women giving birth to singleton infants were included (*n =* 3021). Breastfeeding rates and difficulties according to mode of birth (vaginal, planned c-section and emergency c-section) were compared using cross-tabulations and chi-square tests. A multivariable logistic regression model was created to examine the association between mode of birth on breastfeeding duration to 12 weeks postpartum.

**Results:**

More women who delivered by planned c-section had no intention to breastfeed or did not initiate breastfeeding (7.4 % and 4.3 % respectively), when compared to women with vaginal births (3.4 % and 1.8 %, respectively) and emergency c-section (2.7 % and 2.5 %, respectively). Women who delivered by emergency c-section were found to have a higher proportion of breastfeeding difficulties (41 %), and used more resources before (67 %) and after (58 %) leaving the hospital, when compared to vaginal delivery (29 %, 40 %, and 52 %, respectively) or planned c-sections (33 %, 49 %, and 41 %, respectively). Women who delivered with a planned c-section were more likely (OR = 1.61; 95 % CI: 1.14, 2.26; *p =* 0.014) to discontinue breastfeeding before 12 weeks postpartum compared to those who delivered vaginally, controlling for income, education, parity, preterm birth, maternal physical and mental health, ethnicity and breastfeeding difficulties.

**Conclusions:**

We found that when controlling for socio-demographic and labor and delivery characteristics, planned c-section is associated with early breastfeeding cessation. Anticipatory guidance around breastfeeding could be provided to women considering a planned c-section. As well, additional supportive care could be made available to lactating women with emergency c-sections, within the first 24 hours post birth and throughout the early postpartum period.

**Electronic supplementary material:**

The online version of this article (doi:10.1186/s12884-016-0876-1) contains supplementary material, which is available to authorized users.

## Background

The rate of caesarean section (c-section) in Canada has increased from 17.6 % in 1995 to 27.1 % in 2012 [1]. Although worldwide consensus on the optimal rate of c-section is variable [2], the World Health Organization (WHO) [3] suggests 5–15 % of all live births, far below current c-section rates in Canada. C-sections are associated with poor maternal and infant health outcomes [4–6], such as higher incidences of maternal infection and uterine haemorrhage, and infant respiratory distress and hypoglycaemia [6, 7]; all of which may adversely impact breastfeeding success.

Researchers have shown that women who deliver by c-section delivery are less likely to breastfeed, or delay breastfeeding initiation [8–14]. Breastfeeding within the first hour post-delivery has been cited as an important predictor of continued breastfeeding [15–17]. An early study by Rowe-Murray and Fisher (2002), found that babies born via c-section were less likely to be have skin-to-skin contact immediately after birth and were more likely not to have attempted breastfeeding within the first 24 h post delivery [8]. Skin-to-skin contact has been suggested to improve breastfeeding initiation, maintenance and duration [18]. Delays in breastfeeding initiation accompanying c-section delivery are associated with maternal/infant separation, reduced suckling ability, decreased infant receptivity, and insufficient milk supply, which are predictive of shortened breastfeeding duration [8, 12–14, 19–23].

Breastfeeding is associated with infant health benefits, such as fewer childhood illnesses, lower blood pressure and cholesterol levels, lower prevalence of obesity, and improved intelligence as adults [24, 25]. Maternal benefits of breastfeeding include faster involution of the uterus and lower risk of haemorrhage after birth, in addition to lower a lower lifetime incidence of type II diabetes, and breast and ovarian cancer [26]. These studies have suggested that the benefits of breastfeeding act in a dose response relationship, whereby increasing breastfeeding duration results in more infant health benefits [24–26]. Since it has been established that breastfeeding confers several long-term maternal and infant benefits, it is important to understand the impact of c-section delivery on breastfeeding initiation, and duration.

Health Canada [27] and the WHO [16] recommend that all infants should be breastfed exclusively for the first six months of life, and continue breastfeeding up to and beyond two years of age. Perinatal Health Indicators for Canada (2013) state that breastfeeding initiation rates in Canada remain at approximately 87 % (2005–2010) [28]. However, the 2008 Perinatal Health Report found that only 53.9 % of babies were still breastfeeding at 6 months of age [29]. The rate of exclusive breastfeeding at 6 months has slowly increased from 20.3 % in 2005 to 25.9 % in 2010 [28],but the rate is still low. Only three of the 13 provinces and territories in Canada have designated facilities that meet the WHO Baby Friendly Hospital Initiative (BFI) guidelines [30]; thus, improvements in this area could help to meet the national and international breastfeeding recommendations [16, 27].

Research comparing the effect of c-section delivery to vaginal delivery on breastfeeding duration document conflicting results and often lack adequate sample sizes to support their findings [20, 31–33]. Pérez-Escamilla et al. (1996) indicated that c-section delivery did not affect breastfeeding duration if women initiated breastfeeding from birth and maintained breastfeeding for at least four weeks postpartum, [32]. However, Brown & Jordan (2013) contradict these results reporting that mothers who gave birth via c-section were more likely to discontinue breastfeeding compared to those who gave birth vaginally [33]. The Ontario Mother and Infant Study, by Watt et al (2012), found no differences between c-section and vaginal deliveries on breastfeeding initiation or duration to 6 weeks postpartum [14]. However, this study found that emergency c-sections and assisted vaginal deliveries were associated with an increased likelihood of initiation and duration of breastfeeding, when compared to planned c-section and unassisted vaginal birth [14]. Watt et al. (2012) acknowledged that these results were unexpected and suggested further research to explore all modes of delivery and impact on breastfeeding [14].

In addition, studies have found that the type of c-section, either emergency or planned, influences breastfeeding outcomes [3, 14, 22, 34]. A meta-analysis performed by Prior et al. (2012) pooling 48 studies (*n =* 553,306) found that women who delivered by planned c-section were less likely to initiate any breastfeeding; whereas emergency c-section delivery had no significant impact on initiation. The authors found no association between any type of c-section delivery and exclusive breastfeeding up to six months [31]. However, the authors of this meta-analysis acknowledge that the clarity of the association between mode of birth and several breastfeeding outcomes is limited given that it was not the primary outcome of most of the studies included and that there was evidence of heterogeneity between studies, often resulting from small sample sizes and unadjusted point estimates from individual studies.

Guidelines by the WHO BFI [16] state that keeping mother and baby together for at least the first hour post birth leads to improved initiation and duration of breastfeeding [34, 35]. Although findings support that both emergency and planned c-section affect breastfeeding initiation and duration, the direction of the association among studies is inconclusive and warrants further exploration of breastfeeding, stratified by type of c-section. Our large population-based study examined the effect of planned or emergency c-section and vaginal delivery of singletons, comparing breastfeeding initiation, duration, and difficulties in the early postpartum period, while taking into account for key indicators of breastfeeding success.

## Methods

### Study design

The All Our Babies (AOB) study is a prospective community-based pregnancy cohort in Calgary, Alberta, that began in 2008. Participants completed questionnaires before 25 weeks gestation, 34–36 weeks gestation, and at approximately 4 months (12–16 weeks) postpartum (questionnaires provided in Additional file 1). Further details about the design and methods of the AOB study have been previously published [36]. The AOB collected information during pregnancy on participant’s pregnancy history, health service utilization, demographics, lifestyle, mental, psychosocial and physical health, life events, quality of life, work status, and breastfeeding. Questions about the mode of birth and breastfeeding intention were collected from the pregnancy questionnaire at 34–36 weeks gestation and assessment of breastfeeding outcomes was collected in the questionnaire completed between 12–16 weeks postpartum. Only women giving birth to a singleton infant were included in this study (*n =* 3021). The AOB study was approved by the Child Health Research Office and the Conjoint Health Research Ethics Board of the Faculties of Medicine, Nursing, and Kinesiology at the University of Calgary. Informed consent was obtained from the study participants at the time of recruitment, who were also provided copies for their records.

### Exposure and outcome variables

The main exposure variable of mode of birth was based on maternal self-reported response to the question “How was your baby delivered?” Responses were classified into three levels: emergency c-section (including both before and after the onset of labour), planned c-section, or vaginal delivery. The AOB study validated that maternal recall of the type of birthing method was high when compared to the details in the medical charts [37]. Since medical charts in Calgary and the AOB questionnaire do not distinguish between different types of vaginal delivery, spontaneous, assisted, and instrumental vaginal births were classified together. Breastfeeding outcomes were characterized by maternal reported breastfeeding intention, initiation (e.g., on first attempt, within the first 24 h, prior to leaving the hospital), difficulties (e.g., latching, reports of sore nipples, perceived lack of milk supply), and lactation support sought after discharge. Breastfeeding success was based upon self-reports by breastfeeding mothers in response to two questions: “Were you able to successfully breastfeed on your first attempt?” and “Were you able to breastfeed before you went home from the hospital?” Breastfeeding duration was categorized based on maternal reports of “any breastfeeding” between birth and the data collection time point at 12–16 weeks postpartum. According to breastfeeding status at this time point, we characterized women either as having breastfed for greater than 12 weeks or as having breastfed for 12 weeks or less (early cessation). As the questionnaire asked mothers about any breastfeeding at this time point, we were unable to distinguish between exclusive breastfeeding, or if babies were feeding at the breast versus being offered breast milk with a bottle.

### Data analysis

All data were analyzed using the Statistical Package for Social Science (SPSS Inc. Version 19.0, Chicago, Illinois, USA). Descriptive statistics (means, standard deviation (SD), frequencies, and percentages) were used to characterize the sample and describe breastfeeding and labour and delivery variables. Bivariate associations between demographic, breastfeeding, and labour and delivery variables and mode of birth (vaginal, planned c-section and emergency c-section) were examined using cross-tabulations and Pearson chi square tests.

Multivariable logistic regression was used to examine the association between mode of birth and dichotomized breastfeeding duration, controlling for confounding variables. Significance of covariates was assessed via backwards selection, with comparison to the crude model to assess for confounding whereby all covariates with a *p*-value of <0.05 were retained in the final adjusted model. Potential confounders were assessed in the multivariable model and included: income (<$60,000 or ≥ $60,000), education (≤ high school or at least some post-secondary), previous birth (yes or no), preterm birth (yes or no), postpartum maternal physical health (SF-12 physical component summary score), postpartum maternal mental health (SF-12 mental component summary score), ethnicity (Caucasian or non-Caucasian, breastfeeding difficulties (≤1 difficulty or > 1 difficulty). Only participants with available data were included in the analyses.

This research adhered to the STROBE guidelines for observational studies [38] (checklist added as Additional file 2).

## Results

Table 1 shows demographic and labour and delivery characteristics of the sample of women with singleton infants (*n* = 3021). Of these, 2279 (75 %) delivered vaginally and 739 (25 %) delivered via c-section.Surgical deliveries included 15 % (*n* = 438) emergency c-sections, and 10 % (*n* = 301) planned c-sections. The mean age of the sample was 32 years (SD: 4.4). The majority of the women in our sample were married, highly educated, Caucasian and born in Canada.

**Table 1:** Comparison of demographic and labour and delivery characteristics between vaginal deliveries, emergency c-sections and planned c-sections

Characteristic	Full Sample *N =* 3021^1^	Vaginal *N =* 2279^a^	Emergency c-section *N =* 438^a^	Planned c-section *N =* 301^a^	*P*-value
	*n* (%)	*n* (%)	*n* (%)	*n* (%)	
Demographics					
Maternal age at delivery					
Mean (SD)	31.7 (4.4)	31.5 (4.4)	31.8 (4.5)	33.3 (4.4)	< 0.001
Marital status					
Married/Common Law	2855 (95.3)	2150 (95.2)	409 (93.6)	293 (98.7)	0.006
Other	141 (4.7)	109 (4.8)	28 (6.4)	4 (1.3)	
Education					
High school or less	297 (9.9)	222 (9.8)	44 (10.1)	30 (10.1)	0.998
Some or completed university/college	2233 (74.5)	1683 (74.5)	327 (74.8)	222 (74.5)	
Some or completed grad school	467 (15.6)	354 (15.7)	66 (15.1)	46 (15.4)	
Ethnicity					
Caucasian	2385 (79.6)	1822 (80.6)	327 (75.0)	233 (78.7)	0.026
Non-Caucasian	610 (20.4)	438 (19.4)	109 (25.0)	63 (21.3)	
Income					
< $60,000	471 (16.2)	352 (16.1)	80 (18.8)	39 (13.6)	0.164
≥ $60,000	2434 (83.8)	1838 (83.9)	345 (81.2)	248 (86.4)	
Born in Canada					
Yes	2368 (78.9)	1809 (80.0)	323 (73.9)	233 (78.2)	0.017
No	632 (21.1)	453 (20.0)	114 (26.1)	65 (21.8)	
Labour and delivery					
Parity					
No previous births	1458 (48.9)	1094 (48.6)	304 (69.9)	60 (20.3)	< 0.001
≥1 previous birth	1524 (51.1)	1155 (51.4)	131 (30.1)	235 (79.7)	
Gestational age					
< 37 weeks	211 (7.0)	146 (6.5)	55 (12.7)	10 (3.4)	< 0.001
≥ 37 weeks	2784 (93.0)	2117 (93.5)	379 (87.3)	285 (96.6)	
Small for gestational age^b^					
Yes	300 (10.6)	227 (10.5)	51 (12.6)	22 (8.0)	0.152
No	2537 (89.4)	1927 (89.5)	353 (87.4)	254 (92.0)	

^a^Due to missing values, the denominator changes across variables

^b^Small for Gestational Age defined as <10^th^ percentile for weight-for-gestational age [47]

Significant associations were found across mode of birth compared to parity (*p*<0.001) and gestational age (*p*<0.001). Of the emergency c-section births, approximately 70 % (*n* = 304) were nulliparous compared to 49 % *n* = 1094) of vaginal births and 20 % (*n* = 60) of planned c-sections. Conversely, the majority of women who delivered by planned c-section (80 %, *n* = 235) were multiparous. Although 93 % (*n* = 2784) of the sample delivered at full term (≥ 37 weeks gestation), preterm deliveries were found in 13 % (*n* = 55) of women who delivered by emergency c-section, versus a smaller proportion of vaginal births (6.5 %, *n* = 146) and planned c-section (3.4 %, *n* = 10).

Breastfeeding outcomes according to mode of birth are summarized in Table 2. The majority of the sample, regardless of delivery mode, intended to breastfeed (96 %, *n* = 2846) and initiated breastfeeding (98 %, *n* = 2954). Mode of birth was significantly associated with both planning to breastfeed (*p* = 0.003) and breastfeeding initiation (*p* = 0.012). Women who had a planned c-section, had a higher percentage of those who did not plan to or initiate breastfeeding (7.4 % and 4.3 %, respectively).

**Table 2:** Comparison of breastfeeding success and outcomes between vaginal deliveries, emergency c-sections and planned c-sections

Characteristic	Vaginal *N =* 2279^a^	Emergency c-section *N =* 438^a^	Planned c-section *N =* 301^a^	*P* -value
	*n* (%)	*n* (%)	*n* (%)	
Planned to breastfeed				
Yes	2157 (96.6)	415 (96.3)	274 (92.6)	0.003
No	75 (3.4)	16 (3.7)	22 (7.4)	
Initiated breastfeeding				
Yes	2239 (98.2)	427 (97.5)	288 (95.7)	0.012
No	40 (1.8)	11 (2.5)	13 (4.3)	
Breastfed within 24 h				
Yes	2158(96.9)	392(92.7)	282(97.9)	<0.001
No	69(3.1)	31(7.3)	6(2.1)	
Successful first attempt				
Yes	1677(75.6)	256(60.6)	214(74.3)	<0.001
No	542(24.4)	167(39.4)	74(25.7)	
Successful when leaving hospital				
Yes	1908(90.7)	343(82.7)	263(92.3)	<0.001
No	196(9.3)	72(17.3)	22(7.7)	
Seen LC before leaving hospital				
Yes	862 (40.0)	284 (67.1)	141 (49.3)	<0.001
No	1294 (60.0)	139 (32.9)	145 (50.7)	
Sought additional support after leaving hospital				
Yes	1138 (51.9)	245 (57.8)	117 (40.6)	<0.001
No	1055 (48.1)	179 (42.2)	171 (59.4)	
Difficulties with baby (baby having trouble latching or having a sleepy baby)				
Yes	1016 (44.6)	236 (53.9)	135 (44.9)	0.002
No	1263 (55.4)	202 (46.1)	166 (55.1)	
Discomfort (swollen breasts, sore nipples, or painful breasts)				
Yes	1468 (64.4)	245 (55.9)	182 (60.5)	0.002
No	811 (35.6)	193 (44.1)	119 (39.5)	
Other difficulties (not producing enough milk, flat or inverted nipples)				
Yes	658 (28.9)	180 (41.1)	98 (32.6)	<0.001
No	1621 (71.1)	258 (58.9)	203 (67.4)	

^a^Due to missing values, the denominator changes across variables

Our study found associations between mode of birth and a successful first attempt at breastfeeding (*p*<0.001), breastfeeding within the first 24 h (*p*<0.001), and success with breastfeeding when leaving the hospital (*p*<0.001). Almost 40 % (*n* = 167) of women who had an emergency c-section were unable to successfully breastfeed their baby on the first attempt compared to approximately 25 % of women who either delivered vaginally (*n* = 542) or by planned c-section (*n* = *n* = 74). A higher proportion of women who delivered by emergency c-section did not successfully breastfeed within the first 24 h of birth (7.3 %, *n* = 31), when compared to vaginal births (3.1 %, *n* = 69) and planned c-sections (2.1 %, *n* = 6). Of the women who had an emergency c-section, 7.3 % (*n* = 31) reported not being able to successfully breastfeed within the first 24 h post delivery, and 17 % (*n* = 72) of the women were less likely to breastfeed prior to being discharged from the hospital.

Of the women who initiated breastfeeding (*n* = 2954), 62 % (*n* = 1832) reported having more than one difficulty with breastfeeding. For the women in our study, there were significant differences across mode of birth and breastfeeding difficulties with baby (e.g., latching or a sleepy baby) (*p* = 0.002), personal discomfort with breastfeeding (e.g., sore nipples, swollen breasts) (*p*<0.001), and other difficulties (e.g., perceived low milk supply or having flat or inverted nipples) (*p*<0.001). More than half of the women (54 %, *n* = 236) who delivered by emergency c-section, reported having had breastfeeding difficulties with baby and 41 % (*n* = 180) had described other difficulties. Conversely, approximately 45 % of women who delivered either vaginally (n=1016) or by planned c-section (*n* = 135) reported difficulties with baby and only around 30 % (vaginally *n* = 658 or planned c-section *n* = 98) reported other difficulties with breastfeeding.

The women in our sample were asked about breastfeeding supports accessed in and out of the hospital. Mode of birth was associated with seeing a lactation consultant in hospital (*p*<0.001) and seeking additional support from a breastfeeding clinic or visiting a health care provider after discharge (*p*<0.001). Approximately 67 % (*n* = 284) of women who delivered by emergency c-section saw a lactation consultant and 58 % (*n* = 245) found additional supportive care after discharge. Conversely, about 41 % (*n* = 117) of women who had a planned c-section sought additional breastfeeding support upon discharge from the hospital.

Eighty-three percent (*n* = 2391) of women were still breastfeeding at >12 weeks postpartum. Women who had an emergency or planned c-section were more likely to have stopped breastfeeding at 12 weeks (*p* = 0.02). A multivariable logistic regression model (Table 3) was created to assess breastfeeding duration up to 12 completed weeks postpartum adjusting for income, education, parity, preterm birth, postpartum maternal physical and mental health, ethnicity and number of breastfeeding difficulties. Using vaginal delivery as the reference group, mode of birth remained a significant independent predictor for breastfeeding cessation at or prior to 12 weeks postpartum (*p* = 0.014).

**Table 3:** Unadjusted and adjusted logistic regression model of mode of delivery on breastfeeding duration to 12-weeks postpartum

Independent variable	Unadjusted OR (95 % CI)	Adjusted OR (95 % CI)
Mode of birth^a^		-- ^b^
Emergency caesarean	1.35 (1.03, 1.76)^b^	1.22 (.91, 1.62)^a^
Planned caesarean	1.33 (0.97, 1.82)^a^	1.61 (1.14, 2.26)^b^
Low income	1.61 (1.25, 2.06)^c^	1.58 (1.19, 2.09)^b^
Lower education	2.14 (1.61, 2.85)^c^	1.82 (1.31, 2.53)^c^
No previous birth	1.38 (1.13, 1.68)^b^	1.42 (1.13, 1.77)^b^
Preterm birth	1.66 (1.18, 2.33)^b^	1.54 (1.06, 2.23)^b^
Maternal physical health	0.96 (0.95, 0.98)^c^	.96 (0.94, 0.98)^c^
Maternal mental health	0.99 (0.98, 1.00)^b^	.99 (0.97, 0.99)^b^
Caucasian	1.49 (1.14, 1.95)^b^	1.67 (1.25, 2.22)^c^
≥ 1Breastfeeding difficulty	2.09 (1.67, 2.61)^c^	1.82 (1.43, 2.31)^c^

^a^≥0.05; ^b^<0.05;^c^<0.001

^a^Reference group set as vaginal delivery

In an unadjusted analysis, women who delivered by emergency c-section were more likely to discontinue breastfeeding ≤12 weeks postpartum (OR=1.35; 95 % CI: 1.03, 1.76; *p* = 0.029), when compared to those who delivered vaginally; there was no significant difference between planned c-sections and vaginal birth. However, in the adjusted multivariable logistic regression model (Table 3), women who had a planned c-section were more likely to have early cessation of breastfeeding (≤12 weeks) (OR=1.61; 95 % CI: 1.14, 2.26; *p* = 0.006) when compared to those who delivered vaginally. There was no significant difference in breastfeeding cessation between women who had an emergency c-section and women who delivered vaginally in the adjusted analysis. Covariate patterns and resulting confounding structure in the multivariable model explains the change in magnitude of the effect estimates and the reversal in the association between the levels of exposure in mode of birth in the unadjusted versus adjusted analysis. An important confounder in the adjusted model was parity as it was strongly associated with both early breastfeeding cessation and mode of birth (Table 1 and Table 3); a significantly higher proportion of multiparous women were found among those who delivered by planned c-section (80 %, *n* = 235), compared to vaginal (51 %, *n* = 1155)) or emergency deliveries (30 %, n=131) (*p*<0.0001).

## Discussion

Our findings demonstrate that planned c-sections are associated with reduced breastfeeding success in the first 4 postpartum months, when compared to vaginal births. In particular, we have successfully added to the growing evidence that planned c-sections negatively affect breastfeeding initiation and duration.

All of the Calgary area hospitals support breastfeeding but may not have a specific breastfeeding protocol instituted, nor are they BFI accredited. Each hospital in Calgary has a lactation consultant and post discharge breastfeeding resources with physician and nursing support available. In our study, women who delivered via c-section were more likely to report breastfeeding difficulties when compared to women having had vaginal deliveries. Brown and Jordan (2013) found similar findings; women in their study who delivered via c-section also had more problems with latching, positioning and more pain when compared to those women with vaginal birth [33].

In our study, women who had an emergency c-section were more likely to have had an unsuccessful first breastfeeding attempt, were unable to breastfed their baby within the first 24 h, and were unable to breastfeed upon leaving the hospital. Zanardo et al. (2010) found similar results, reporting that women who delivered via emergency c-section were more likely not to have been able to breastfeed their baby at delivery or at discharge [39]. It has been hypothesized that the maternal and fetal stress response associated with complications during delivery, particularly related to c-sections, may be related to increased difficulty and early cessation in breastfeeding during the early postpartum period [14, 33]. Lactogenesis may be equally affected by the insult of abdominal surgery in both planned and emergency c-sections, although the notion of an “emergency” may invoke a greater or prolonged maternal stress response.

Our data showed that women with planned c-sections were more likely to indicate they did not intend to breastfeed and therefore did not initiate it compared to those with other delivery modes. This concurs with a large meta-analysis that found an association between a lower likelihood of initiation of breastfeeding and planned c-sections [31]. Although our study did not analyze maternal or medical reasons for planned c-section, reasons for planned c-section coupled with no intention to breastfeed should be explored given that the rate of planned c-sections has been rising since the year 2000 [1, 28]. The Canadian Maternity Experiences Survey in 2008 reported that of all 6,421 women who completed the survey, 26 % delivered by c-section and 13.5 % of all c-sections were planned [29]. Of those planned c-sections, the majority (86.9 %) were planned for medically indicated reasons [29]. The Canadian Perinatal Surveillance System acknowledged that c-section rates are increasing and recognize that c-sections on maternal request could be part of this growing burden [21]. However, a study by Hutton and Kornelsen (2012) found that in British Columbia, one of the provinces with the highest rates of c-section delivery, very few women requested c-sections with no medical indication and concluded that the increased rate of c-section delivery in Canada may not be related to maternal choice [40]. Similarly, a review of 17 articles on the topic of maternal decision making found that only a small portion of women requested c-sections for non-medically indicated reasons in each study [41]. However, the studies included in this review were susceptible to a high risk of bias due to small sample sizes, warranting further studies specifically investigating maternal decision making surrounding requested c-section deliveries for non-medical indication.

Lower breastfeeding initiation and increased difficulties with breastfeeding in women with c-section deliveries may be related to a physiologic influence on lactogenesis [42, 43]. A study by Evans et al. (2003) found that breast milk transfer among women with c-sections was significantly lower in the first 5 days postpartum, compared to women with vaginal births [42]. Similarly, Scott and Binns (2007) found that delayed onset of lactation was significantly higher in mothers that delivered via c-section compared to those that delivered vaginally [43]. It is postulated that the hormonal pathway that stimulates lactogenesis is disrupted by c-section delivery, either from maternal stress or decreased oxytocin secretion, and can hinder milk production [34, 39, 42, 44]. Late preterm infants, those born between 34 and 36 6/7 weeks gestation, often have a multitude of issues including disorganized sucking skills, lower birth weight and low level of alertness, all of which can adversely affect breastfeeding initiation [34, 45]. Since planned c-sections are performed prior to the onset of labour and before 40 weeks gestation, it is possible that physiological reasons are responsible for the decreased likelihood or troubled breastfeeding initiation. In response, the American College of Obstetricians and Gynecologists (2013) recommended that c-sections performed prior to onset of labour should not be conducted prior to 39 weeks unless medically indicated [46].

When controlling for other variables potentially associated with breastfeeding, we found that planned c- section predicts early breastfeeding cessation, defined as weaning at or prior to 12 weeks. Although Zanardo et al. (2013) found that c-section delivery was associated with lower rates of exclusive breastfeeding at 6 months, no difference between planned versus emergency c-sections was found [19]. Women who had planned c-sections in our study were less likely to intend to breastfeed or initiate breastfeeding, and less likely to seek lactation support. Although we controlled for parity in our multivariable model, the bivariate associations with mode of birth and breastfeeding intention and initiation may be related to the other factors such as variable infant feeding choices or return to work. Multiparous women may have had a previous challenging breastfeeding experience and chose not to breastfeed. Previous breastfeeding experiences in women are known to impact future decisions surrounding breastfeeding. Although this study did not look at prior experiences with breastfeeding, women who have had difficult breastfeeding experiences with prior births may require particular attention to promote future positive breastfeeding experiences. More targeted interventions towards women planning on c-section delivery from the perinatal period and into the postpartum time, may encourage breastfeeding initiation and continuation of breastfeeding.

As our study found that women who have emergency c-sections were more likely to have difficulties with breastfeeding and to seek additional lactation support, focused education surrounding breastfeeding techniques and encouragement to these mothers could be given immediately post birth in order to provide the appropriate anticipatory guidance to reduce difficulties. In Canada, there are few hospitals that have breastfeeding practices that adhere to these standards set by the BFI. We recommend that health care professionals should be aware that those women who have delivered via c-section may require more supportive care immediately post-delivery and in the early postpartum period. In particular, our study identified that those who delivered via emergency c-section may require access to lactation consultation and other supports for breastfeeding. These supports could be made available in the immediate post-partum period in order to encourage breastfeeding within 1 h post birth and ensure successful breastfeeding within the first 24 h, as recommended by the BFI and many research studies as being indicative of long term breastfeeding success [15–17]. As women who delivered via emergency c-section were more likely not to have successfully breastfed before leaving the hospital, the postpartum home visit presents an ideal opportunity to provide additional breastfeeding assessments and support to overcome any difficulties for these women.

### Limitations

Our study reflects the demographics of urban Calgary (predominately caucasion, highly educated and of a higher SES); however, generalizability to other populations should be interpreted with this in mind. Due to the nature of the questionnaire, the outcome variable in this study looked at maternal self-reports of any breastfeeding and did not explore the relationship between mode of birth and exclusive breastfeeding. As the majority of the variables used in these analyses were assessed via questionnaire at 4 months, we were unable to assess variations in health status over the entire postpartum period. It would be interesting to further explore how variations in maternal physical or mental health are associated with each mode of birth and if there is a relationship with breastfeeding. Additionally, there could be some residual confounding related to the obstetric factors that we were unable to control for given content contained in the questionnaire. In particular, further analysis, through medical chart review, looking at how the complications during labour and delivery could impact and possibly confound the relationship between mode of birth and breastfeeding success and duration. Since mode of birth was based on maternal self-report, it is possible that there was misclassification between emergency and planned deliveries. However, a recent validation study conducted by the AOB confirmed that maternal recall of delivery method (vaginal, emergency c-section, or planned c-section) was high when compared to electronic medical chart records [37]. Also, this study did not distinguish between the type of vaginal birth (assisted versus spontaneous), and some instrumental deliveries can be challenging. This would attenuate responses and underestimate the positive effect that an uncomplicated vaginal delivery has on breastfeeding initiation, and duration.

## Conclusion

Our study suggests that c-sections are associated with more breastfeeding difficulties, greater use of resources, and shorter breastfeeding duration compared to vaginal deliveries. Planned c-sections coupled with no intention of breastfeeding may be an emerging trend among women that requires further exploration. As planned c-section delivery may be associated with lower intention to and initiate breastfeeding as well as early cessation of breastfeeding, health care provider support and counselling in order to provide anticipatory guidance surrounding breastfeeding could be given prenatally to pregnant mothers and families considering a planned c-section. As well, additional supportive care made available to lactating women with emergency c-sections during the immediate to early postpartum period would be recommended in order to ensure early success with breastfeeding.

## Additional files

Additional file 1: Questionnaires. (DOCX 230 kb) Additional file 2: Checklist. (DOC 89 kb)

## Abbreviations

AOB: all our babies
c-section: caesarean section
WHO: World Health Organization
