# Supplementary material for: The impact of caesarean section on breastfeeding initiation, duration and difficulties in the first four months postpartum
Source: BMC Pregnancy Childbirth. 2016 Apr 26;16:90. doi: 10.1186/s12884-016-0876-1 (PMC4847344; doi:10.1186/s12884-016-0876-1)
Supplement: Additional file 1: — Questionnaires. (DOCX 230 kb) [file 12884_2016_876_MOESM1_ESM.docx]

675516353

**Less than 24 weeks Questionnaire**


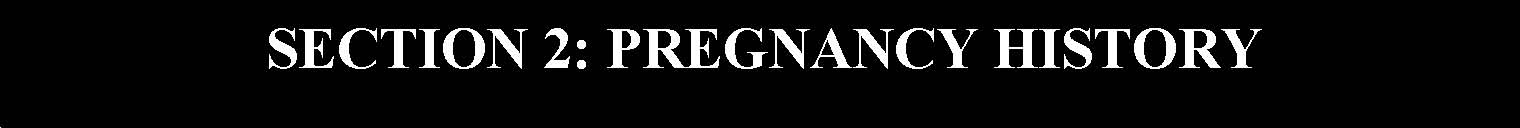


**1. Have you ever been pregnant before? [prevpreg]**

1 -
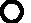
Yes

2 -
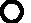
No If no, skip to question 11.

**2. How many times have you been pregnant (not including this pregnancy)?**
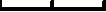
 **[numpreg]**


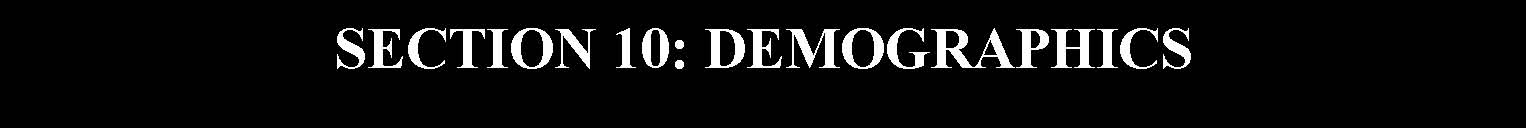


**1. How would you describe your current marital status? [mstat]**

1 -
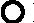
Single 5 -
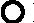
Divorced

2 -
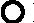
Single with partner 6 -
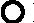
Separated

3 -
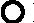
Married 7 -
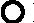
Widowed

4 -
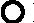
Common law

**2. What is your birth date?**
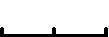
/
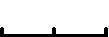
/
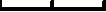

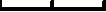

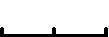


**MM DD YYYY [bday]**

**3. What is the highest level of education you have completed? [educ]**

1 -
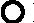
Some Elementary or High School (Grades 1 - 12)

2 -
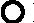
Graduated High School

3 -
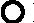
Some college, trade, university

4 -
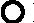
Graduated college, trade, university

5 -
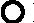
Some graduate school

6 -
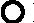
Completed graduate school

**4. Were you born in Canada? [born]**

1 -
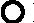
Yes

2 -
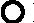
No **If no, which country were you born in?** **[country]**_______________

**How long have you lived in Canada?**
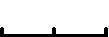
 months OR
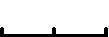
 years

**[mthcan] [yrcan]**

**What was your status upon entering Canada? [statcan]**

2 -
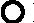
Immigrant 1 -
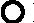
Dual Citizen

3 -
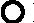
Refugee 4 -
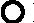
Other: ­**[otherstatcan]**______________

**5. How long have you lived in Calgary or the surrounding area?**
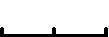
 months OR
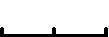
 years  **[mthcal] [yrcal]**

**6. How would you describe your ethnic background? [eth]**

1 -
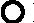
White / Caucasian 9 -
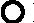
Filipino

2 -
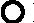
Black / African North American 10 -
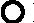
Latin American

3 -
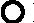
First Nations person registered 11 -
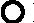
Southeast Asian

(under the Indian Act of Canada)

4 -
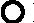
First Nations person not registered 12 -
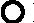
Arab

5 -
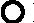
Inuit 13 -
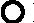
West Asian

6 -
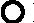
Métis 14 -
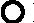
Korean

7 -
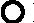
Chinese 15 -
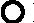
Japanese

8 -
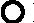
South Asian 16 -
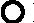
Mixed / Other: **[othereth]** ________________

**16. What is the total income, before taxes and deductions, of all household members from all sources in the past 12 months?** Your best guess is ok. **[income]**

1 -
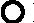
Less than $10,000

2 -
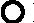
$10,000 -$19,999

3 -
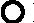
$20,000 -$29,999

4 -
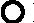
$30,000 -$39,999

5 -
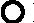
$40,000 -$49,999

6 -
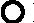
$50,000 -$59,999

7 -
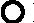
$60,000 -$69,999

8 -
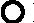
$70,000 -$79,999

9 -
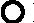
$80,000 -$89,999

10 -
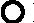
$90,000 -$99,999

11 -
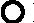
$100,000 or more

**18. Please enter today's date.
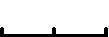
/
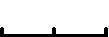
/**
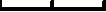

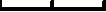
**
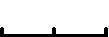
 [q1atoday]**

**MM DD YYYY**

**Third Trimester Questionnaire**


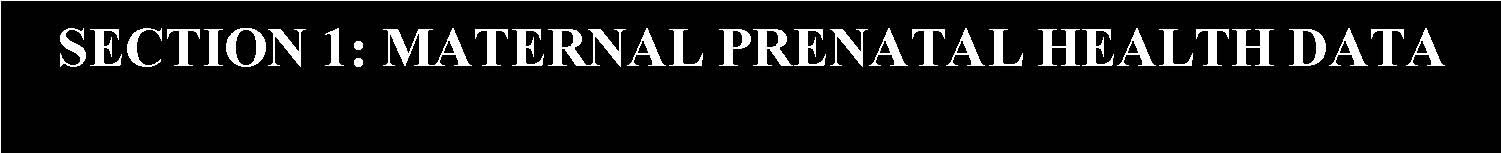


**17. Are you planning to breastfeed this baby? [q2brstfeed]**

1 -
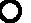
Yes 2 -
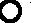
 No 3 -
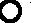
 Unsure

**If yes, for how many weeks are you planning to breastfeed your baby?**
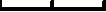

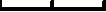
 weeks **[q2wksbrstfeed]**

(e.g. 1 month is approximately 4 weeks)

**Four-Month Postpartum Questionnaire**

| **SECTION 1: YOUR NEW BABY (BABIES)** |
| --- |
|  |
|  |

***We would like to begin by asking about your delivery and the last few weeks of your pregnancy. When we***

***use the words "this pregnancy", we mean your recent pregnancy during which you participated in this study.***

***All questions are about your recent pregnancy or delivery only.***

**1. How many babies did you deliver in this pregnancy?**
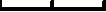
 baby/ babies **[q3numbabies]**

**2. How many weeks pregnant were you when your baby/ babies was/were born?**
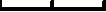
 weeks **[q3ga]**

For each of the following questions, please provide the information for each of the children born from your most recent delivery only. **Please leave all non‐applicable questions blank** *(ie. if you had 1 baby, leave information for Baby #2 and #3 blank)*.

BABY 1: 1 - Boy
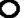
 **2 -** Girl
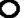
 **[q3gender1]** Birthdate:
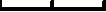
 /
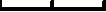
 /
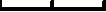

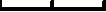
 **[q3bday1]**

MM DD YYYY

Birthweight:
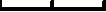
 lbs
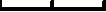
 oz OR
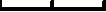

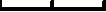
 g

**[q3bwlb1] [q3bwoz1] [q3bwkg1]**

BABY 2: 1 - Boy
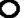
 **2 -** Girl
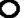
 **[q3gender2]** Birthdate:
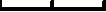
 /
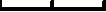
 /
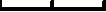

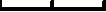
 **[q3bday2]**

MM DD YYYY

Birthweight:
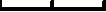
 lbs
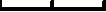
 oz OR
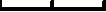

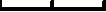
 g

**[q3bwlb2] [q3bwoz2] [q3bwkg2]**

**3. How was your new baby (babies) delivered? [q3delivery]**

1 -
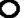
Vaginally

2 -
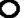
You went into labour but had an emergency cesarean section (c-section)

3 -
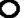
You did not go into labour and had an emergency cesarean section (c-section)

4 -
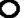
You had a planned cesarean section.

**4. Was your labour induced? In other words, did a doctor or nurse give you a medication or apply a gel to speed up the start of your labour? [q3induced]**

1 -
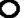
 Yes 2 -
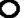
 No

**5. Did you have any medication to manage pain during your delivery? [q3painmgmt]**

1 -
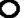
 Yes 2 - No

**If yes, which of the following were you given? *(check all that apply)*** 0 IF NOT SELECTED

1 - Epidural **[q3epidural]**

1 - Laughing gas (nitrous oxide) **[q3gas]**

1 - Tens (trans electronic nerve stimulator) **[q3tens]**

1 - Morphine or Demerol **[q3morphine]**

1 - Other **[q3painmedoth]**: ____________**________ [q3othpainmgmt]**

**6. Did you have anyone to support you at your delivery other than the medical staff? [q3suplabour]**

1 - Yes 2 - No

**If yes, please select all of the people who were present to support you during your delivery** 0 IF NOT SELECTED

1 - Partner **[q3suplabpart]** 1 - Friend **[q3suplabfriend]**

1 - Mother **[q3suplabmom]** 1 - Doula **[q3suplabdoula]**

1 - Sister **[q3suplabsis]** 1 - Other **[q3suplaboth]**: ________**[q3othsuplabour]**

**7. Where did you deliver your baby/babies? [q3hospital]**

1 - Foothills Hospital

2 - Peter Lougheed Hospital

3 - Rocky View General Hospital

4 - At home

5 - Other: **[q3hospitaloth]**_______________

***If you did not deliver in a hospital, please skip to section 2: Breastfeeding*SECTION**

**8. After your baby was born, how long was their hospital stay? Please note, this question is asking about your baby/babies, not the time you spent in the hospital or during delivery.**

Hours **[q3babystayhrs]** OR Days **[q3babystayday]**

**9. After your baby was born, how long was your hospital stay?**

Hours **[q3momstayhrs]** OR Days **[q3momstayday]**

**10. On a scale of 1 to 10 how ready did you feel to go home from the hospital after your delivery? (1 being not at all ready and 10 being completely ready) [q3homeready]**

1 - 1 2 - 2 3 - 3 4 - 4 5 - 5 6 - 6 7 - 7 8 - 8 9 - 9 10 - 10

| **SECTION 2: BREASTFEEDING** |
| --- |
|  |
|  |

**1. Did you breastfeed or feed breast milk to your baby, even if only for a short time? [q3bfinitiate]**

1 - Yes

2 - No

**If no, what was your main reason for not breastfeeding? *(please select only one)* [q3whynobrstfd]**

1 - Went back to work or school

2 - Afraid it would hurt

3 - No support from family/baby's father

4 - Embarrassed

5 - Just couldn't imagine breastfeeding my baby

6 - Didn't think I could make enough milk or good enough milk

7 - Was worried about diet or medications that might hurt my baby

8 - Told by a doctor or midwife that I should not breastfeed my baby

9 - Other: **[q3whynobfoth]**___________________________________________

**2. How do you feel about the amount of time you had to talk with your healthcare providers about breastfeeding while you were pregnant? [q3brstfeedinfo]**

1 - Too much time

2 - Just the right amount

3 - Not enough time

***If you did not breastfeed your new baby at any time, please skip to Question 18***

**3. As a result of breastfeeding your baby, have you experienced any of the following? *(please check all that apply)***

0 IF NOT SELECTED

1 - Difficulties with the baby such as the baby having trouble latching or having a sleepy baby **[q3bfdiff1]**

1 - Discomfort such as swollen breasts, sore nipples, or painful breasts **[q3bfdiff2]**

1 - Difficulty breastfeeding such as not producing enough milk, or having flat or inverted nipples **[q3bfdiff3]**

1 - Been tired or fatigued **[q3bfdiff4]**

1 - Any other challenge **[q3bfdiffoth]** Please specify: **[q3bfdiffoth2]**___________________________________________

_______________________________________________________

**4. Have you experienced your partner or family members being unsupportive of breastfeeding? [q3unsupbrstfeed]**

1 - All of the time 2 - Most of the time 3 - Some of the time 4 - A little of the time 5 - None of the time

**5. Has breastfeeding been inconvenient for you? [q3bfinconvenient]**

1 - All of the time 2 - Most of the time 3 - Some of the time 4 - A little of the time 5 - None of the time

**6. Was your first attempt at breastfeeding your baby within 24 hours of giving birth? [q3bf24hr]**

1- Yes 2- No

**If no, how many hours or days after you gave birth did you start breastfeeding or expressing breastmilk?**

Hours **[q3bfhrs}**  OR Days **[q3bfdays]**

**7. Were you able to successfully breastfeed on your first attempt? [q3bfsuccess]**

1 - Yes 2 - No

**8. Were you able to breastfeed before you went home from the hospital? [q3ablebfhosp]**

1 - Yes 2 - No

**9. Did you see a lactation consultant before you went home from the hospital? [q3lactconsult]**

1 - Yes 2 - No

**10. Since leaving the hospital, have you sought any additional breastfeeding support? [q3addsupbf]**

1 - Yes **If yes, where did you receive this support? [q3addsupbf2]_________________________________**

2 - No

**11. If you delivered outside of a hospital, have you sought any additional breastfeeding support? [q3outsidebfsup]**

1 - Yes **If yes, where did you receive this support? [q3_outsidebfsup_where]________________________**

2 - No

**12. When you started breastfeeding, how difficult was it for you physically? [q3bfphys]**

***(1 being extremely difficult, 10 being not at all difficult)***

1 - 1 2 - 2 3 - 3 4 - 4 5 - 5 6 - 6 7 - 7 8 - 8 9 - 9 10 - 10

**13. When you started breastfeeding, how difficult was it for you emotionally? [q3bfemo]**

***(1 being extremely difficult, 10 being not at all difficult)***

1 - 1 2 - 2 3 - 3 4 - 4 5 - 5 6 - 6 7 - 7 8 - 8 9 - 9 10 - 10

**14. In the first week, what best describes what your baby was fed? [q3feedwk1]**

1 - Only breast milk

2 - Mostly breast milk but with formula

3 - Mostly formula with breast milk

4 - Only formula

**15. In the past week, what best describes what your baby was fed? [q3babyfednow]**

1 - Only breast milk

2 - Mostly breast milk but with formula

3 - Mostly formula with breast milk

4 - Only formula

**16. Have you started feeding your baby solid foods? [q3solidfood]**

1 - Yes 2 - No

**If yes, at what age did you start feeding your baby solid foods?**  weeks months **[q3wksolidfood]** **[q3mthsolidfood]**

**17. Have you started giving your baby juice? [q3juice]**

1 - Yes 2 - No

**If yes, at what age did you start feeding your baby juice?**  weeks months

**[q3wkjuice] [q3mthjuice]**

**18. Are you still breastfeeding your baby? [q3stillbf]**

1 - Yes 2 - No

**If no, how long did you breastfeed your baby?**  weeks months

**[q3brstfdwks] [q3brstfdmths]**

**If no, what was the main reason you stopped breastfeeding? *(please select only one)* [q3bfstop]**

1 - Not enough milk 9 - Advice of partner or family member

2 - Tired / Fatigued 10 - Advice of doctor or nurse

3 - Sleepy baby 11 - Preferred formula feeding

4 - Discomfort 12 - I wanted to drink alcohol

5 - Illness: Self 13 - I was having problems with it

6 - Illness: Child 14 - I did not enjoy it

7 - Child weaned himself/herself 15 - I planned to stop breastfeeding the baby

8 - Returned to school/work 16 - Other: **[q3bfstopoth]**________________________

**33. Overall, your breastfeeding experience has been: [q3overallbfexp]**

1 - Very positive

2 - Positive

3 - Neither negative nor positive

4 - Negative

5 - Very negative
